# General health survey in patients with small abdominal aortic aneurysm

**DOI:** 10.3389/fcvm.2025.1600775

**Published:** 2025-08-19

**Authors:** A. Arnautovic, H. Dalyanoglu, L. J. Vallejo Castano, W. Garabet, J. Mulorz, A. Knapsis, S. Düsing, I. N. Schellinger, H. Schelzig, U. Raaz, M. Duran, M. U. Wagenhäuser

**Affiliations:** ^1^ Department of Vascular and Endovascular Surgery, Medical Faculty and University Hospital Duesseldorf, Heinrich-Heine-University, Duesseldorf, Germany; ^2^ Department of Cardiac Surgery, Medical Faculty and University Hospital Duesseldorf, Heinrich-Heine-University, Duesseldorf, Germany; ^3^ Department of Angiology, University Medical Center Leipzig, Leipzig University, Leipzig, Germany; ^4^ Molecular and Translational Vascular Medicine, Department of Cardiology and Pneumology, Heart Center at the University Medical Center Göttingen, Göttingen, Germany; ^5^ Partner Site Göttingen, German Center for Cardiovascular Research e.V., Göttingen, Germany; ^6^ Department of Vascular and Endovascular Surgery, Marienhospital Gelsenkirchen, Teaching Hospital of Ruhr-University Bochum, Gelsenkirchen, Germany

**Keywords:** quality of life, abdominal aortic aneurysm, diagnosis, survey, small aortic aneurysms

## Abstract

**Background:**

Abdominal aortic aneurysm (AAA) is a dynamic disease characterized by a continuous diameter progression. AAA may therefore be categorized in early-stage disease (small diameter AAA) and late-stage disease (large diameter AAA). To date, there is no effective therapy for patients in early stages, resulting in disease-specific stressors. This study was designed to quantify these stressors using a specifically designed questionnaire.

**Methods:**

A self-designed 17-item questionnaire with categorical response options was distributed to patients with small AAA. The aim of the approach was to gain a deeper understanding of patients' quality of life (QoL), psychological state and attitudes towards hypothetical future treatment options.

**Results:**

122 patients were contacted, 63 of whom consented to participate in the study. The study cohort was predominantly older than 65 years, 84% were male and 56% had been diagnosed with small-size AAA less than 1 year ago. In summary, the AAA diagnosis has a significant impact on all aspects of personal health perception, including physical, social and mental health. The overall QoL was found to be impaired compared to the situation before the AAA diagnosis for most of the respondents, and the current treatment options were considered unsatisfactory. The majority of small AAA patients surveyed would be open to new (invasive) therapies to stop disease progression.

**Conclusion:**

The diagnosis of AAA leads to a reduction in QoL, which impairs the physical, social and mental health, particularly affecting mental health. Patients would welcome treatment options in early stages of their disease.

## Introduction

Abdominal aortic aneurysm (AAA) is defined by a dilation of the aorta that is greater than 3 cm or an increase in diameter exceeding 50% compared to the aortic diameter at the level of the diaphragm (1). The prevalence of the disease in individuals over the age of 50 is estimated to be as high as 7.9% in men and 1.3% in women which symbolizes a significant health burden worldwide (2, 3). AAA are commonly classified by size into early- and late-stage disease, with early-stage referring to small aneurysms measuring less than 50 mm in women and less than 55 mm in men.). As of today, there is no treatment available at early stages of the disease which effectively prevents the diameter progression. Late-stage disease is characterized by larger aneurysms exceeding these sex-specific thresholds, for which elective surgical repair is generally recommended in asymptomatic patients due to the markedly increased risk of rupture (4). In case of rupture, high mortality rates are observed and even postintervention mortality is significant (5).

There are different therapeutic options available: endovascular aortic repair (EVAR) and open surgical repair (OSR). EVAR now demonstrates high procedural quality and improved outcomes due to continuous technical and technological advancements, making it the most commonly used therapeutic option for AAA (6). However, despite significant improvements in patient safety, the long-term complication rate following EVAR is estimated to range between 16% and 30%. Here, the most common complications include both endograft device-related and systemic complications (7). Among them, different types of endoleak (EL) may occur, which may warrant re-interventions exposing affected patients to the risk of recurrent endovascular procedures.

AAA represents a dynamic pathological condition that remains asymptomatic during its early stages. The annual expansion rate of the aneurysm varies between 0.09 cm and 0.46 cm, contingent upon the initial aneurysm size and other contributory risk factors (6). The recommended intervals for follow-up ultrasound—widely regarded as the modality of choice for AAA surveillance due to its safety, accessibility, and cost-effectiveness—are informed by these clinical considerations and range from 6 months to several years, depending on the current aneurysm diameter (4). Such fact heightens patient awareness, marked by specific stress factors, particularly related to the anticipated enlargement of the AAA diameter. Quality of life (QoL), as well as changes within its individual dimensions following a diagnosis of AAA, appear to be subject to disease-specific alterations, likely attributable to the initially asymptomatic course of the condition. That being said, the recent literature has indicated a reduction in overall health status among patients with AAA; however, the validated questionnaires employed neither comprehensively capture all individual dimensions of health nor are they specifically designed to address potential disease-specific stressors associated with AAA (8–12). Furthermore, it has been shown that the impact on QoL in AAA patients under surveillance (up to 90% of all incidentally detected AAA) is highly individual, with a notable influence on emotional parameters (13).

This study seeks to contribute to the existing body of knowledge by identifying disease-specific stressors in the early stages of AAA through the administration of a custom-designed, AAA-specific questionnaire to patients with small aneurysms who do not yet meet the criteria for therapeutic intervention. Additionally, it seeks to evaluate the potential readiness of AAA patients for treatment during the initial phase of the disease.

## Methods

Building upon the Short-Form Health Survey (SF-8), a newly developed 17-item questionnaire specifically designed to assess AAA-related stressors was administered to patients diagnosed with small abdominal aortic aneurysms not yet requiring intervention. Recruitment took place at the Clinic for Cardiac Surgery and the Clinic for Vascular and Endovascular Surgery at University Hospital Düsseldorf, Germany, as well as at the Clinic for Vascular and Endovascular Surgery at Sankt Marien-Hospital Buer and Marienhospital Gelsenkirchen, Gelsenkirchen, Germany (see Supplementary Figure 1). Here, all patients who were diagnosed with a small AAA between January 1, 2023, and June 1, 2024, were included in the study. The timeframe was chosen to keep the potential recall period for patients within a reasonable and reliable timeframe. No exclusion criteria applied. Patients were mailed the survey form together with an unmarked return envelope.

The questionnaire was divided into three sections: a demographic part, a health-specific part, and a part directed to the standard of care. Patients completed the questions independently, without external assistance. The analysis was conducted anonymously. The questionnaire aimed to address three key questions for patients with small AAAs, as follows:

1. How do patients feel about being diagnosed with small-size AAA at an early stage, particularly considering the current lack of treatment options at this stage?
2. To what extent does the diagnosis of a small AAA affect the patient's daily life? Are there any restrictions or changes in everyday life since the diagnosis?
3. How would patients react to the hypothetical possibility of an early, minimally invasive treatment for AAA? Would they opt for such treatment if it was available?

The study was approved by the ethic Committee of the Medical Faculty at the Heinrich-Heine-University Düsseldorf (study number: 2024-2815). As this study was fully anonymous, informed consent was waived.

Categorical data in a pre-diagnosis vs. post-diagnosis comparison were analyzed using Fisher's exact test to evaluate shifts resulting from the AAA diagnosis. The level of significance was set to *p* < 0.05.

## Results

A total of 122 patients with small AAAs were contacted, with 63 patients ultimately participating, resulting in a response rate of 52%. The standardized Cronbach's alpha for the QoL–related items of the questionnaire was 0.84, indicating a high level of internal consistency (see Supplementary Figure 1).

The first three questions assessed demographic data of the study cohort. The majority of patients were older than 65 years, male, smokers and were diagnosed with a small AAA less than one year ago (Table 1).

**Table 1:** Patient demographic characteristics and key data. Data are presented as absolute and relative frequencies, *n* (%), for categorical variables within the study cohort, including age, gender, smoking status, and time since AAA diagnosis. (*n* = 63).

Demographic characteristics and key data		Number of patients (*n*) and Percentage (%)	
		*n* = 63	%
Age	18–64 years	12	19
	≥64 years	50	79.4
	Missing data	1	1.6
Gender	Male	53	84.1
	Female	9	14.3
	Missing data	1	1.6
Smoking status	Smoking	23	36.5
	Quit smoking	26	41.3
	Never smoked	12	19
	Missing data	2	3.2
Time since AAA diagnosis	<1 years	34	54
	1–5 years	19	30.2
	>5 years	7	11.1
	Missing data	3	4.8

**Figure 1:**
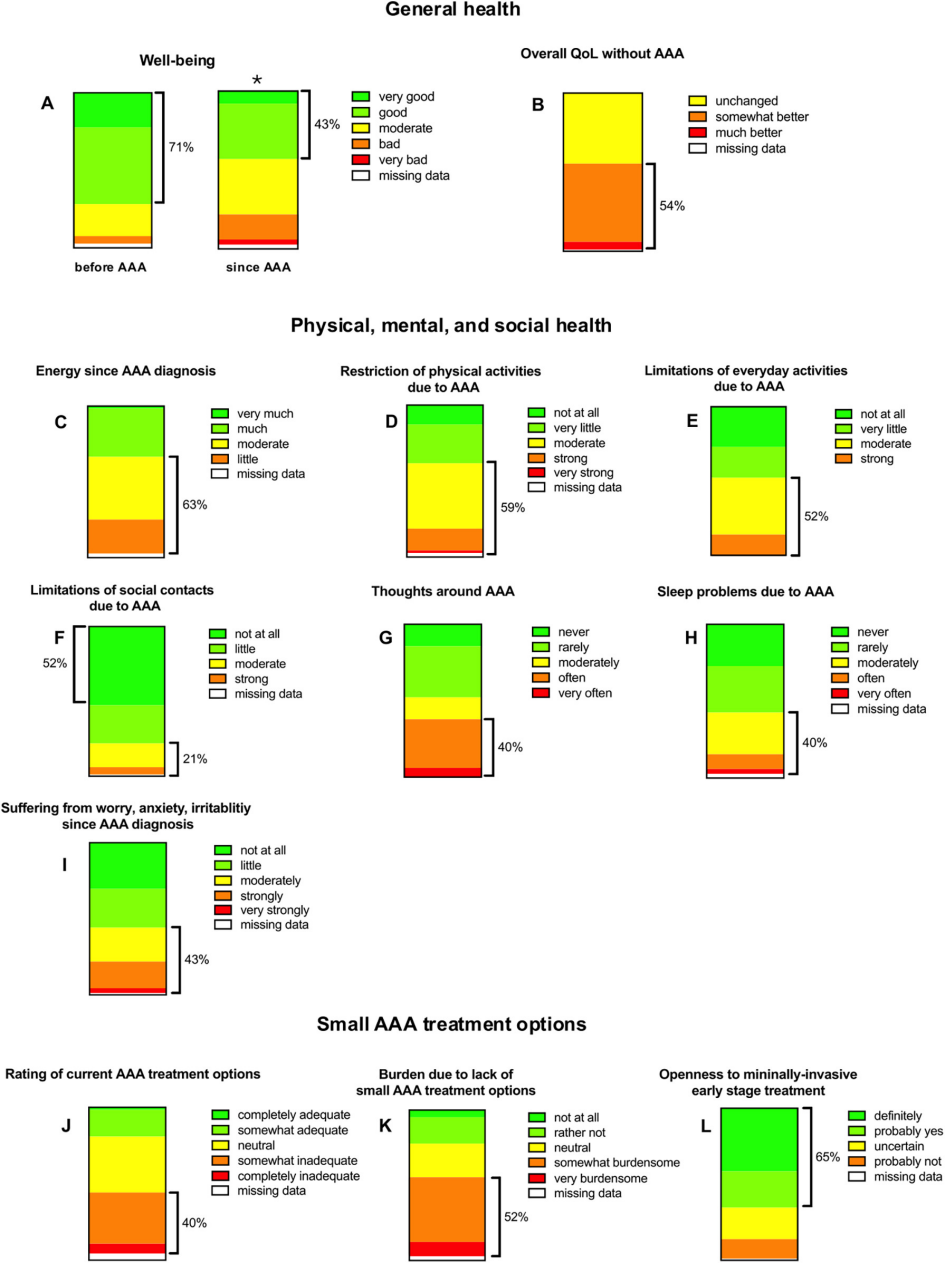
Patient-reported outcomes (charts A–L) regarding general health **(A–B)**, physical, mental, and social well-being **(C–I)**, and perspectives on treatment options for small abdominal aortic aneurysms (AAA) **(J–L)**. Each chart reflects response distributions across ordinal scales (e.g., from very good to very bad). Responses reflect perceived well-being, energy levels, activity limitations, psychological burden, and openness to future treatments. While 43% of participants reported improved well-being since diagnosis, 65% expressed willingness to consider minimally invasive early-stage interventions. *Indicates *p* < 0.05 vs. well-being status before AAA.

The first question of the health-related section addressed personal well-being before and after receiving the diagnosis. Here, the diagnosis of a small AAA was associated with a statistically significant decline in personal well-being (Figure 1A and Table 2) (*p* < 0.05). Specifically, the ratio of patients with good and very good personal well-being dropped from 71% before to 43% after diagnosis. Conversely, the proportion of individuals who rated their personal well-being as bad or very bad almost quadrupled, rising from 5% to 19% after the diagnosis of an AAA (Figure 1A and Table 2). The decline in personal well-being also translated in corresponding findings in the quality of life (QoL) assessment. Here, 54% of the patients would find their quality of life at least somewhat better without AAA diagnosis (Figure 1B and Table 2).

**Table 2:** Absolute and relative frequencies.

General well-being **before** AAA diagnosis	Number of patients (%)	General well-being **after** AAA diagnosis	Number of patients (%)
Very good	14 (22.2)		5 (7.9)
Good	31 (49.2)		22 (34.9)
Moderate	13 (20.6)		22 (34.9)
Bad	3 (4.8)		10 (15.9)
Very bad	0		2 (3.2)
Missing data	2 (3.2)		2 (3.2)
Overall QoL without AAA	Number of patients (%)		
Much worse	0		
Somewhat worse	0		
Unchanged	28 (44.4)		
Somewhat better	31 (49.2)		
Much better	3 (4.8)		
Missing data	1 (1.6)		
Energy since AAA diagnosis	Number of patients (%)		
Very much	1 (1.6)		
Much	20 (31.8)		
Moderate	26 (41.3)		
Little	14 (22.2)		
No energy at all	0		
Missing data	2 (3.2)		
Reduction of physical activities	Number of patients (%)		
Not at all	17 (26.9)		
Very little	13 (20.6)		
Moderate	24 (38.1)		
Strong	9 (14.3)		
Very strong	0		
Missing data	0		
Limitation of social contacts	Number of patients (%)		
Not at all	33 (52.4)		
Little	16 (25.4)		
Moderate	10 (15.9)		
Strong	3 (4.8)		
Very strong	0		
Thoughts on AAA	Number of patients (%)		
Never	9 (14.3)		
Rarely	21 (33.3)		
Moderately	9 (14.3)		
Often	20 (31.8)		
Very often	4 (6.4)		
Missing data	0		
Sleep problems due to AAA	Number of patients (%)		
Never	17 (26.9)		
Rarely	19 (30.2)		
Moderately	17 (26.9)		
Often	6 (9.5)		
Very often	2 (3.2)		
Missing data	2 (3.2)		
Suffering from worry, anxiety irritability since AAA diagnosis	Number of patients (%)		
Not at all	19 (30.2)		
Little	16 (25.4)		
Moderately	14 (22.2)		
Strongly	11 (17.5)		
Very strongly	2 (3.2)		
Missing data	1 (1.6)		
Rating of current AAA treatment options	Number of patients (%)		
Completely adequate	1 (1.6)		
Somewhat adequate	11 (17.5)		
Neutral	23 (36.5)		
Somewhat inadequate	21 (33.)		
Completely inadequate	4 (6.4)		
Missing data	3 (4.8)		
Burden due to lack of small AAA treatment options	Number of patients (%)		
Not at all	3 (4.8)		
Rather not	11 (17.5)		
Neutral	14 (22.2)		
Somewhat burdensome	27 (42.9)		
Very burdensome	6 (9.5)		
Missing data	2 (3.2)		
Openness to minimal invasive early-stage treatment options	Number of patients (%)		
Definitely	26 (41.3)		
Probably yes	15 23.8)		
Uncertain	13 (20.6)		
Probably not	8 (12.7)		
Definitely not	0		
Missing data	1 (1.6)		

The table presents both the absolute frequencies (*n*) and relative percentages (%) of patient responses for each questionnaire item and corresponding answer option (*n* = 63).

To identify specific factors driving QoL deterioration, we analyzed items of physical, mental and social health. 63% of the patients reported on self-perceived energy levels that were moderate and worse since AAA diagnosis (Figure 1C and Table 2). Also, the majority of patients reported at least moderately restricted physical activities since diagnosis (Figures 1D,E and Table 2). Specifically, 59% of the patients reduced sports at least moderately (Figure 1D and Table 2), and 52% patients reduced everyday activities (e.g., household tasks, shopping, etc.) to an at least to a moderate extent (Figure 1E and Table 2). Interestingly, for most patients (52%) there were no restrictions in contact with family members. Yet, 21% of the patients reported at least moderate limitation regarding social contacts (Figure 1F and Table 2). When examining mental health, we observed that most patients' thoughts are frequently centered on their illness. Interestingly, only 13% of patients reported never thinking about their AAA, while 40% stated that their thoughts often or very often revolve around the disease during the day (Figure 1G and Table 2). For many patients, the diagnosis of a small size AAA means having difficulties falling asleep: 40% of the patients experienced this issue at least moderately and in 13% of the patients this even occurred often to very often (Figure 1H and Table 2). Of note, the severity of worries and anxiety related to AAA disease exhibits a similar distribution pattern (Figure 1I and Table 2).

The final three questions addressed current treatment options and aimed to assess patients' willingness to adopt a hypothetical early-stage treatment. Notably, 40% of respondents felt that existing treatment options and therapeutic approaches are somewhat or completely inadequate (Figure 1J and Table 2). This sentiment is particularly driven by the lack of options for small AAAs, which are managed conservatively through surveillance—a method perceived as rather or very burdensome by 52% of patients (Figure 1K and Table 2). Consequently, the willingness to pursue treatment in the early stages of the disease is remarkably high. If a minimally invasive treatment capable of slowing AAA growth were available, 65% of respondents indicated they would opt for it (Figure 1L and Table 2).

## Discussion

Given the limitations of a 52% response rate and a modest sample size of 63 patients, this study suggests that the diagnosis of small, early-stage AAA represents a significant and widespread health burden, adversely affecting patients' physical, social, and psychological well-being.

The World Health Organization defines quality of life (QoL) as an individual's perception of their place in life, considering the cultural and value systems they live within, as well as their personal goals, expectations, standards, and concerns (14). Given the progressive nature of AAA, which may be asymptomatic in its early stages but still exposes the patient to a constant, progressive life-threatening risk of rupture, a small AAA diagnosis impacts nearly every dimension of QoL as defined by the WHO (15, 16). In this context, the reduced QoL suggested by the findings of the present study cohort is not only logical but also understandable.

Analyzing different QoL dimensions in detail, our survey suggests a significant impact on the mental health of patients with small-size AAA. Recent qualitative studies describe the mixed emotions patients experience following an AAA disease, ranging from relief at having a diagnosis to fears about disease progression. Here, both Botero et al. and Hansson et al. found that patients valued the detection of their AAA, while the diagnosis also led to worry, uncertainty, and feelings of anxiety (17, 18). Mental health has a significant impact on the vasculature, with conditions like depression, anxiety, psychological distress, and post-traumatic stress disorder recognized as independent cardiovascular risk factors (19). Interestingly, several mechanistic associations have already been further elucidated. For instance, it has been demonstrated that mental stress can induce transient peripheral endothelial dysfunction, which is considered as an early-stage phenomenon of atherosclerosis (20). Moreover, additional cardiovascular parameters appear to be influenced by mental stress. Specifically, the impact on mean arterial pressure is believed to be mechanistically mediated by elevated levels of circulating neurotransmitters, as well as by interactions with vascular inflammation through increased concentrations of pro-inflammatory cytokines (21, 22). However, the reverse relationship, i.e., the extent to which cardiovascular diseases, or the mere knowledge of their existence, influence mental health, appears to be far less well documented. In their review of 11 studies, Ericsson et al. addressed the question to what extent the knowledge of having an aneurysm may be worrying and burdensome for affected patients. In analogy with our data, the authors found that patients diagnosed with AAA tend to have inferior QoL and health than those without such diagnosis (23). Focusing on mental health in particular, a study from Kim et al. reported an increased rate of depression rate upon AAA diagnosis predominantly at younger ages (24). In this context, individual disease perception—and its positive modification by others—as well as social support within the close social circle of the AAA patient, may serve as important protective factors. However, the presence and influence of these factors were beyond the scope of the current questionnaire-based study and may rather serve as a basis for future research initiatives aiming to improve preventive patient care. That being said, the question arises as to the clinical implications for treating physicians. In light of the present data, early psychological support for patients in the initial stages of AAA disease may prove particularly beneficial and could therefore be considered as a potential component of patient care.

In addition to mental health, an AAA diagnosis also appears to severely restrict everyday activities. Our data may suggest a substantial limitation in self-perceived energy levels as well as in the ability to perform daily activities following an AAA diagnosis. This is consistent with findings by Bath et al., who reported that physical health limitations were the most persistent restriction after an AAA diagnosis, while the impact on mental QoL was more transient (25). As a matter of fact, the loss of self-perceived energy levels cannot be directly attributed to AAA, since it is not a consumptive disease. There is likely to be a significant overlap with other dimensions of health, such as mental and social factors. This interwovenness and interdependency of the individual dimensions of health is not novel but has already been introduced to the current literature (26).

Social health is another area potentially influenced by a AAA diagnosis. It is noteworthy that the present study did not identify a significant association between AAA and restrictions in social activities. Similarly, a Swedish cross-sectional study by Damhus et al. also found that social relationships might remain relatively unaffected by an AAA diagnosis, with participants reporting no changes in their contact with family members (27). In fact, social support may play a crucial role in helping patients cope with the stress and worry associated with AAA.

Understandably, following an AAA diagnosis, many patients seek ways to positively influence the course of the disease (28). The relatively high participation rate in this study, despite the lack of direct physician involvement, underscores how deeply concerned patients are about their condition and how much it affects their lives. In this context, considerable emphasis should be placed on the findings pertaining to patients’ willingness to adopt a hypothetical therapeutic intervention, even during the early stages of abdominal aortic aneurysm (AAA) disease. In view of patients’ evident preference for minimally invasive interventions—particularly when considering factors related to procedural burden and postoperative recovery—such therapeutic modalities may prove especially appropriate and accessible for individuals in the early stages of AAA disease (29, 30). Given the hypothetical nature of the question posed to respondents in this study, the reported high willingness to undergo minimally invasive treatment should be interpreted exploratory in nature and does not warrant immediate changes in clinical treatment strategies. Should such an objective be pursued in future studies, more detailed, procedure-specific risk–benefit information would be essential to facilitate informed, individualized decision-making. Nonetheless, the reported overall willingness to consider minimally invasive therapy during this so-called ‘window of missed opportunity’ reflects the substantial burden the disease imposes on patients’ QoL across multiple dimensions. This also emphasizes to need for healthcare providers to prioritize the development and implementation of effective therapeutic strategies that help patients better manage the disease. Patients appear to recognize the critical need for intervention at this early stage.

This study is subject to several limitations. Firstly, the relatively small sample size, combined with a response rate of 52% and recruitment from a limited number of centers, may limit the representativeness of the study population and introduce a considerable risk of self-selection bias. Consequently, the generalizability of the findings to the broader population may be constrained. Therefore, the presented survey data need validation on larger cohorts in multi center settings and findings should be interpretated with caution. However, the demographic characteristics of our patient population (including sex, age, and smoking status) align with those of the general AAA population, suggesting that the sample may still be representative despite its limited size. Additionally, the questionnaire used was self-designed and has not undergone specific validation, which restricts direct comparisons with the existing literature. As a result of the custom-made design, certain aspects of AAA may not have been fully addressed. Although rigorous measures were undertaken to assess internal consistency through the calculation of Cronbach's alpha, which yielded a high level of internal consistency, the employment of a non-validated questionnaire may impinge upon the reliability and external validity of the results. Lastly an additional potential selection bias may also compromise our reported data since included patients may be more concerned with general health issues due to their AAA diagnosis.

In conclusion, although AAA is often detected at an early, asymptomatic stage, the awareness of a progressive and potentially life-threatening condition may significantly impact patients’ QoL, as well as their physical and mental health. A substantial proportion of patients with small AAA appear to harbor reservations regarding the adequacy of the current standard of care. This may, in turn, reflect a preference for earlier intervention—currently hypothetical—that aims to arrest disease progression.

## Data Availability

The raw data supporting the conclusions of this article will be made available by the authors, without undue reservation.

## Supplementary material

Supplementary Figure 1 Questionnaire assessing quality of life in patients with abdominal aortic aneurysm (AAA). This self-administered questionnaire consists of two sections: The first four items gather general health and demographic information (age, gender, smoking status, and time since diagnosis). The subsequent items (questions 5–17), under the heading “Questionnaire Quality of Life with Abdominal Aortic Aneurysm”, specifically assess physical, emotional, and social aspects of living with an AAA. Patients were instructed to respond solely in relation to their aneurysm, excluding other comorbidities. Responses were collected anonymously and voluntarily. For the calculation of Cronbach’s alpha, only items 5–17 were included, as they directly address disease-specific quality of life.

Supplementary Figure 2 Correlation matrix of AAA Quality of Life (QoL)-associated questionnaire items for reliability assessment. The heatmap illustrates the pairwise Pearson correlation coefficients among questions 5–13 of the custom-made AAA questionnaire for Cronbach's alpha calculation. The color gradient reflects the magnitude and direction of the correlations, providing a graphical overview of inter-item relationships. Cronbach Alpha is .84, suggesting a high internal consistency.
