# Supplementary figures and images for: General health survey in patients with small abdominal aortic aneurysm
Source: Front Cardiovasc Med. 2025 Aug 19;12:1600775. doi: 10.3389/fcvm.2025.1600775 (PMC12404035; doi:10.3389/fcvm.2025.1600775)

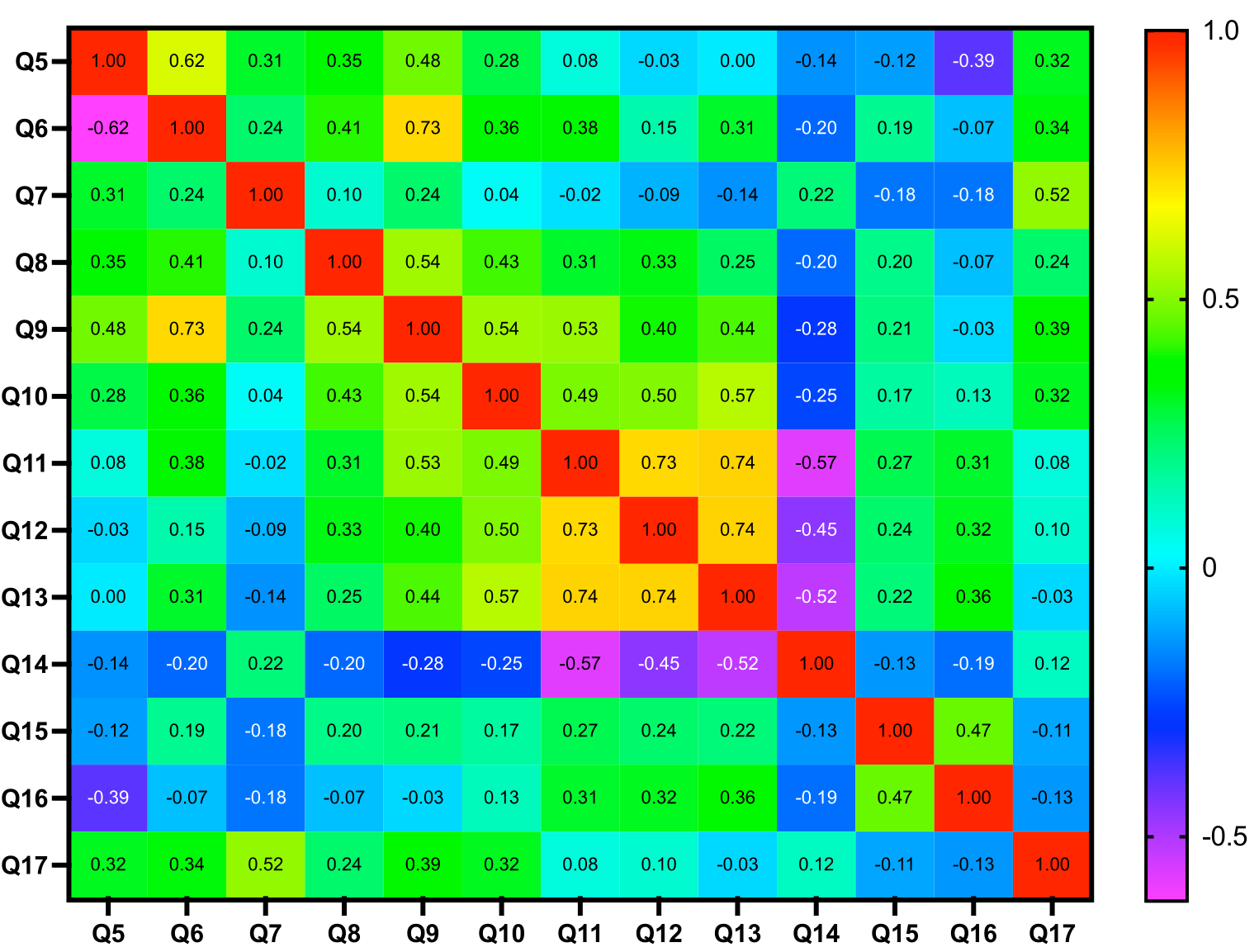

Supplement: Supplementary Figure 1 — Questionnaire assessing quality of life in patients with abdominal aortic aneurysm (AAA). This self-administered questionnaire consists of two sections: The first four items gather general health and demographic information (age, gender, smoking status, and time since diagnosis). The subsequent items (questions 5–17), under the heading “Questionnaire Quality of Life with Abdominal Aortic Aneurysm”, specifically assess physical, emotional, and social aspects of living with an AAA. Patients were instructed to respond solely in relation to their aneurysm, excluding other comorbidities. Responses were collected anonymously and voluntarily. For the calculation of Cronbach’s alpha, only items 5–17 were included, as they directly address disease-specific quality of life. [file Image1.tiff]
